# Carbohydrate Partitioning and Antioxidant Substances Synthesis Clarify the Differences Between Sugarcane Varieties on Facing Low Phosphorus Availability

**DOI:** 10.3389/fpls.2022.888432

**Published:** 2022-05-11

**Authors:** Miriam Büchler Tarumoto, Murilo de Campos, Letusa Momesso, Carlos Antônio Costa do Nascimento, Ariani Garcia, Renata Bruna dos Santos Coscolin, Jorge Martinelli Martello, Carlos Alexandre Costa Crusciol

**Affiliations:** ^1^Department of Crop Science, College of Agricultural Sciences, São Paulo State University (UNESP), Botucatu, Brazil; ^2^Department of Soil Science, Luiz de Queiroz College of Agriculture (ESALQ), University of São Paulo (USP), Piracicaba, Brazil; ^3^Department of Chemistry and Biochemistry, Institute of Biosciences, São Paulo State University (UNESP), Botucatu, Brazil

**Keywords:** sugarcane nutrition, roots, nutrient absorption, P use efficiency, *Saccharum* spp., antioxidant enzymes, carbohydrate partitioning

## Abstract

Phosphorus (P) availability is important for metabolic process, tillering and formation of a vigorous root system in sugarcane, but sugarcane varieties differ in P uptake efficiency. This study evaluated the enzymatic, nutritional, and biometric parameters of two sugarcane varieties under two conditions of P availability by monitoring the initial development of plants grown in nutrient solution. The experiment was performed using randomized complete block design (RCBD) with five replicates and included two varieties, RB966928 (high nutritional requirements) and RB867515 (low nutritional requirements), and two concentrations of P in the nutrient solution: low (2 mg L^−1^) and suitable (16 mg L^−1^). Carbohydrate concentrations and partitioning, leaf nutrient concentrations, enzymatic activity, and shoot and root biometric parameters were analyzed. Regardless of sugarcane variety and the part of the plant, reducing sugar were approximately 32.5% higher in RB867515 and 38.5% higher in RB966928 under suitable P compared with low P. Sucrose concentrations were significantly higher in both varieties under suitable P than in low P. According to PCA, the relationship between reducing sugars and sucrose was closer in RB966928 than in RB867515. Under low P, soluble protein content decreased, and the activities of the antioxidant enzymes superoxide dismutase (SOD), catalase (CAT), and ascorbate peroxidase (APX) and the concentrations of hydrogen peroxide (H_2_O_2_), and malondialdehyde (MDA). The variety RB966928 under suitable P appears to have a high capacity for proline (120%) upregulation under abiotic stress compared with RB867515 (54%), and thus higher biomass accumulation of this RB966928 variety; however, RB867515 had superior results compared to RB966928 under low P. Suitable P increased leaf concentrations of N, P, Mg, B, and Mg and decreased leaf Zn content. Root and shoot dry matter, root length, plant height, and root and stalk diameter increased by suitable P. Regardless of variety, both nutritional and biometric parameters were directly influenced by P levels, including sugarcane yield. In relation of sugarcane dry matter, RB966928 was less sensitive to low P levels and more responsive to P supply than RB867515 and thus may be more suitable for environments in which P is limiting.

## Introduction

Soil phosphorus (P) levels are a major economic and environmental concern worldwide due to the rising price of P fertilizer, which is the result of the growing costs of fossil fuels, mining, processing, transport, and import taxes (Withers et al., 2018; Soltangheisi et al., 2019b; Garske and Ekardt, 2021). P directly impacts plant development, metabolic processes, and crop yield, particularly in soils that are highly weathered, volcanic ash-derived, acidic or P-fixing (Cakmak, 2002; Roy et al., 2017). Due to the low natural fertility of acidic soils, suitable P fertilization management is indispensable to achieve favorable production (Jaiswal et al., 2017), while minimizing negative environmental impacts (Bordonal et al., 2018). P is critical for plant metabolic activity, especially protein synthesis, cell division, photosynthetic processes, energy storage and supply, sugar metabolism, respiration, and sucrose production and transport (Marschner, 2012). In addition, P is a component of essential biomolecules like DNA, RNA, and phospholipids (Plaxton and Lambers, 2015).

In Brazil, sugarcane is of major economic importance due to its uses in biofuel and sugar production and as a source of millions of jobs (Moraes et al., 2015; Cardoso et al., 2018). Restricted soil P availability adversely impacts biomass production and yields and is frequently responsible for low sugarcane production efficiency (Gopalasundaram et al., 2012; Bachiega Zambrosi, 2021). In addition, plants need major quantities of P to improve nutrient uptake strategies and reduce nutrient losses by enhancing P mobility from the bulk soil to rhizosphere (Hamoud et al., 2019; Ning et al., 2019). Consequently, P-based fertilizers and the management of their application in sugarcane crop production have been studied extensively (Cherubin et al., 2016; Borges et al., 2019; Soltangheisi et al., 2019a; Crusciol et al., 2020). P fertilization is generally performed without considering the specific hybrid, even though sugarcane varieties differ in their efficiency of P utilization to maintain adequate growth and yield (Sundara, 1994; Zambrosi et al., 2014) and the rate of sucrose accumulation (Lingle, 1997). Complex hybrid (clone) species of the genus *Saccharum* are nearly exclusively used in sugarcane cultivation to reduce production costs, while increasing productivity. One approach to improving production sustainability, yields, and plant physiological performance is to identify genotypes that ameliorate the effects of P scarcity on sugarcane development (Zambrosi et al., 2014).

Abiotic stresses such as salinity, heat, and nutritional deficiency lead to the accumulation of reactive oxygen species (ROS; You and Chan, 2015; Aleksza et al., 2017; Choudhury et al., 2017; Gao et al., 2020). ROS such as singlet oxygen (O_2_^−^) and hydrogen peroxide (H_2_O_2_) are highly reactive and toxic and lead to damage to proteins, lipids, carbohydrates, and DNA (Gill and Tuteja, 2010). ROS accumulation as a result of environmental stress is a major cause of crop yield reduction (Molinari et al., 2007; Cia et al., 2012; Zidenga et al., 2012). Low P levels can also induce ROS production by compromising the use of light energy harvested by reaction centers and the synthesis of ATP and NADPH (Croft et al., 2017), which results in electron transport chain overload (Singh et al., 2019). Antioxidant molecules such as superoxide dismutase (SOD), catalase (CAT), ascorbate peroxidase (APX), and proline reduce these deleterious effects on plant yield by promoting ROS homeostasis (Decros et al., 2019; Hasanuzzaman et al., 2021). In addition, studies have been shown that abiotic stress can be mitigated by strategies use of plant varieties (He et al., 2017; Li et al., 2019; Sheteiwy et al., 2021; Yang et al., 2021), and adequate varieties face better the environmental stress when have greater metabolism performance to produce these molecules.

The differences in the abilities of sugarcane varieties to use soil P pools and metabolize acquired P are not fully understood. To address this gap, the present study examines the effects of varying conditions of P availability on different sugarcane varieties recommended for the same cropping environment. We hypothesized that the responses of different varieties to low P availability depend on their ability to synthesize antioxidant substances, particularly proline. To test this hypothesis, we monitored the initial development of two sugarcane varieties in nutrient solution under different conditions of P availability. We sought to relate these responses to metabolite biosynthesis and partitioning, enzymatic activity, nutritional content, shoot and root morphological parameters, and P utilization.

## Materials and Methods

### Experimental Setup and Design

The study was carried out in a greenhouse equipped with an internal heating/cooling air circulation system under a climate-controlled environment (temperature between 23 and 32°C with a photoperiod of 14 h of light). The experiment was performed using randomized complete block design (RCBD) and comprised four treatments and five replicates. The treatments comprised two sugarcane varieties (RB966928 and RB867515) and two P concentrations in the nutrient solution, 2 and 16 mg L^−1^, which are considered “low” and “suitable,” respectively, for sugarcane plant development.

### Sugarcane Varieties and P Levels

Sugarcane varieties were selected for this study based on their characteristics (Ridesa, 2010). The variety RB966928 is recommended for cropping environments with medium to high potential and is characterized by excellent germination of the cane plant; excellent ratooning; high tillering of the cane plant and ratoon crop, with excellent inter-row closure; very high crop yield; medium useful period of industrialization (UPI); early ripening; rapid development; and medium sucrose and fiber content; intermediate response to P supply (Ridesa, 2011; da Silveira et al., 2014). The variety RB867515 is characterized by high crop yield; medium to late ripening; good ratooning and inter-row closure; high sucrose and medium fiber content; and medium UPI. Both varieties must be planted in areas with medium natural fertility, and harvesting between July and September is recommended; superior response to P supply (Ridesa, 2011).

Preliminary testing of both varieties with six P levels (0, 2, 4, 8, 16, and 32 mg L^−1^ of P) using KH_2_PO_4_ and H_3_PO_4_ showed that performance was similar in the treatments containing 16 and 32 mg P L^−1^ and that H_3_PO_4_ provided better pH stability of the solution. In this study, P rates tested followed the rates tested by Mata-Alvarez et al. (2000) in soil conditions. Among the low P availability conditions, the treatments containing 2 and 4 mg L^−1^ P had similar performance, whereas plants cultivated under no P application (0 mg L^−1^ P) did not develop after 2 weeks. Based on these results, P concentrations of 2 mg P L^−1^ and 16 mg P L^−1^ using H_3_PO_4_ were defined as “low” and “suitable,” respectively.

### Seedling Establishment, Trial Setup, and Nutrient Solution

Cane-cutting seedlings (length of 3 cm and one vegetative bud) were harvested from a commercial nursery. The buds were extracted from the upper third of each cane cutting to ensure uniform sprouting. For germination, the cane cuttings were placed in plastic trays containing coarse sand and periodically irrigated with deionized water. The trays were placed on benches inside the greenhouse under controlled air humidity. At 27 days after emergence, seedlings were selected according to their sprouting uniformity and then transplanted into nutrient solution. The seedlings were fixed in foam plates and subsequently transferred to a plastic pot so that only the root system was in contact with the nutrient solution. Each pot contained four sugarcane seedlings and was filled with 4 L of nutrient solution under constant aeration. After 51 days, the plants were transferred to larger pots containing 8 L of nutrient solution.

The nutrient solution composition was as follows (in mg L^−1^): 138 N-NO_3_ as Ca(NO_3_)_2_.4H_2_O; 20 N-NH_4_ as NH_4_NO_3_; 141 K as K_2_SO_4_; 151 Ca as Ca(NO_3_)_2_; 17 Mg as MgSO_4_.7H_2_O; 56 S as ZnSO_4_.7H_2_O; 0.04 Cu as CuSO_4_.5H_2_O; 3.6 Fe as Rexolim M48 (6.5% Fe); 0.04 Mn as MnCl_2_.4H_2_O; 0.08 Mo as (NH_4_)_6_Mo_7_O_24_; 0.15 Zn as ZnSO_4_.7H_2_O; 0.003 B as H_3_BO_3_; 33 Cl as KCl; and 2 (low) or 16 (high) P as H_3_PO_4_. To avoid possible interference from saline effects on the initial growth of the seedling roots, the nutrient solution was diluted to one-third ionic strength during the first week and half ionic strength during the second week and replaced with full ionic strength solution during the third week. The maximum volume variation of the solution was 5%, and water lost to evapotranspiration was replaced with deionized water. The pH was monitored daily and maintained within a range of 5.5–6.5 by adjustment with 0.1 M HCl or 0.1 M NaOH solution when necessary. The nutrient solution was replaced with fresh solution weekly.

### Plant Parameters, Root Measurements, and Leaf Nutrient Concentrations

Evaluations were conducted 73 days after the plants were transplanted to nutrient solution. Stalk diameter was measured using a caliper, and a ruler was used to measure both internode length and plant height (from the base to the top of the plant). Tiller number was also determined. Root length and diameter were measured by a digitizer coupled to a computer with WinRhizo software version 3.8-b (Regent Instruments Inc., Quebec, Canada) as described by Tennant (1975). To determine root, shoot, and total dry matter, the plants were divided into roots, stalks, and leaves, washed in running water, dried in paper bags in a forced-air oven at 65°C until reaching a constant weight, and weighed. The middle third of the dry leaves was ground in a Wiley-type mill with a 1-mm screen, and the concentrations of N, P, K, Ca, Mg, S, B, Cu, Zn, Mn, and Fe were determined according to Malavolta et al. (1997).

### Carbohydrates

The partitioning of carbohydrates between roots, stalks, and leaves was determined by measuring the reducing sugar, sucrose, and starch concentrations in the respective dry matter mass. The same milled plant material used to nutritional analysis was subjected to reducing sugars (fructose and glucose) and sucrose fractionation by high-performance liquid chromatography (HPLC) on a Shimadzu model 10A chromatograph with an RID-10A refractive index detector and model LC-10AD isocratic pump (Bossolani et al., 2021). The starch concentration was determined according to Somogyi-Nelson (Nelson, 1944; Somogyi, 1952) and the absorbance was measured in a spectrophotometer at 535 nm.

### Enzymatic Activity and Proline

Leaf samples were ground in liquid nitrogen in a porcelain mortar to obtain extracts for the analysis of SOD, CAT, and APX activities and protein and proline content.

#### Superoxide Dismutase

To measure SOD activity, 300 mg of plant material was ground in 3 ml of 100 mmol L^−1^ potassium phosphate buffer (TFK, pH 6.8) containing 0.1 mmol L^−1^ EDTA, 0.1% (v/v) 2-mercaptoethanol, 0.1% (v/v) Triton X-100, 30 mg of polyvinylpyrrolidone (PVP) and 20 mmol L^−1^ ascorbate. The homogenate was centrifuged at 15,000 *g* for 15 min at 4°C, and the supernatant was collected for further analysis. SOD activity was determined as described by Giannopolitis and Ries (1977). The reaction medium consisted of 52.5 mmol L^−1^ TFK (pH 7.8), 0.1 mmol L^−1^ EDTA, 13 mmol L^−1^ methionine (pH 7.8), 2 mmol L^−1^ riboflavin, and 0.075 mmol L^−1^ nitroblue tetrazolium (NBT). A 10-μl aliquot of enzyme extract was added, and the production of blue formazan from NBT reduction in the presence of light was monitored in a spectrophotometer at 560 nm. The results were expressed in units of SOD mg^−1^ protein, where 1 unit of SOD is the amount of enzyme required to reduce the production of blue formazan by 50%.

#### Catalase

To measure CAT activity, 300 mg of plant material ground in liquid nitrogen was added to 3 ml of a solution consisting of 100 mmol L^−1^ trifluoromethyl ketone (pH 7.0), 2 mmol L^−1^ EDTA, 0.1% (v/v) Triton X100, 0.1% (v/v) 2-mercaptoethanol, 20 mmol L^−1^ ascorbate, and 30 mg of polyvinylpolypyrrolidone (PVPP). After centrifuging the homogenate at 15,000 *g* for 15 min at 4°C, a 20-μl aliquot of the supernatant was added to 3 ml of reaction medium containing 50 mmol L^−1^ TFK (pH 7.0) and 12.5 mmol L^−1^ H_2_O_2_. CAT activity was determined by monitoring the drop in the absorbance of H_2_O_2_ at 280 nm in a spectrophotometer (Peixoto et al., 1999) using the molar extinction coefficient of H_2_O_2_ (e = 39.4 mmol L^−1^ cm^−1^) and expressed as specific CAT activity (mKat).

#### Ascorbate Peroxidase

Ascorbate peroxidase activity was determined by adding a 100-μl aliquot of crude extract to 2.9 ml of 50 mM potassium phosphate buffer, pH 6.0, containing ascorbate and H_2_O_2_ at final concentrations of 0.8 and 1.0 mM, respectively (final volume of 3.0 ml). The decrease in absorbance was monitored at 290 nm, and the specific activity of the enzyme (μKat μg Prot^−1^) was calculated using a molar extinction coefficient of 2.8 mM-1 cm^−1^ (Koshiba, 1993).

#### Hydrogen Peroxide

The concentration of H_2_O_2_ was determined according to the method of Alexieva et al. (2001). Frozen leaf material (0.4 g) was ground in liquid nitrogen using a pestle, and 4 ml of 0.1% trichloroacetic acid (TCA) was added for extraction. The material was centrifuged (12,000 rpm) for 15 min at 4°C, and 200 μl of the supernatant was mixed with 200 μl of 100 mM potassium phosphate buffer (pH 7.5) plus 800 μl of 1 M potassium iodide and incubated at 1°C for 1 h. After warming the samples to room temperature, the absorbance was measured in a spectrophotometer at 390 nm. The concentration of H_2_O_2_ was determined by reference to a standard curve and expressed as μmol g^−1^ FW.

#### Malondialdehyde

Malondialdehyde (MDA, an indicator of lipid peroxidation) was determined according to Heath and Packer (1968). Frozen leaf plant material (0.4 g) was ground in liquid nitrogen using a pestle and suspended in 4 ml of 0.1% (w/v) TCA+ 20% (w/v) PVPP. The homogenate was centrifuged (10,000 rpm) for 15 min at 4°C, and a 250-μl aliquot of the supernatant was mixed with 1 ml of 20% TCA + 0.5% thiobarbituric acid (w/v) solution, heated at 95°C for 30 min, and cooled on ice for 10 min. After centrifugation for 10 min at 10,000 rpm, the absorbance of the supernatant was measured in a spectrophotometer (535 and 600 nm). The results were expressed as nmol MDA g^−1^ FW.

#### Soluble Protein

Soluble protein was determined in triplicate by the method of Bradford (1976) with bovine serum albumin (BSA) as the standard. Leaf extracts were obtained by grinding approximately 500 mg of plant tissue previously frozen in liquid nitrogen in 2 ml of phosphate buffer (0.1 M, pH 6.7) containing PVPP to control the oxidation of the material. After centrifugation for 10 min at 5,000 rpm, the supernatant was used as the crude extract. Three 100-μl aliquots of extract and 5 ml of Bradford’s reagent were pipetted into a glass test tube, and the solution was homogenized by agitation on a shaker. After 15 min, the absorbance at 595 nm was measured in glass cuvettes in a spectrophotometer.

#### Proline

The concentration of proline (μg ml^−1^ extract) was determined using the colorimetric method of Bates et al. (1973). The reaction comprised 100 μl of crude extract, 2 ml of acidic ninhydrin, and 2 ml of glacial acetic acid and was heated in a water bath at approximately 100°C for 60 min. After cooling, the absorbance at 520 nm was determined. A standard curve was constructed using proline concentrations of 0, 20, 40, 60, 80, and 100 mg L^−1^ (p.a.).

### Statistical Analysis

Data were subjected to ANOVA, and means were compared by the least significant difference (LSD) test (*p* ≤ 0.05). Sugarcane variety and P level were considered fixed factors. When there was significant interaction between factors, treatments were combined to improve data visualization of treatment differences. Principal component analysis (PCA) was performed by using the statistical software package from Statistica (Statsoft, 2005). To determine the factors in PCA, the Kaiser rule was used, and factors with eigenvalues > 1 or explaining over 85% of the total variance were considered (Kaiser, 1958). Correlations > |0.70| were considered (Manly, 1994).

## Results

### Carbohydrate Partitioning

**Figure 1:**
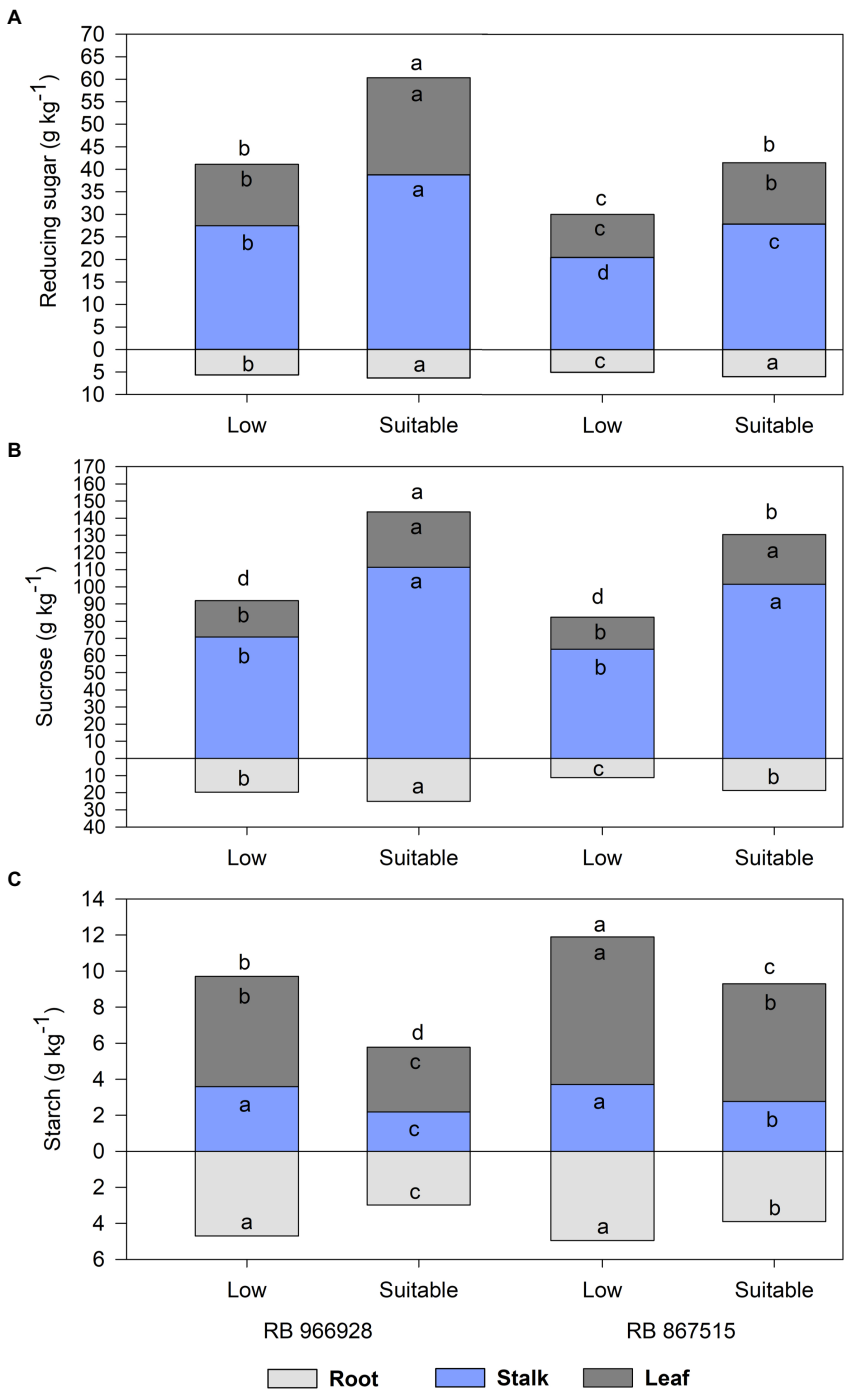
Carbohydrate partitioning into **(A)** reducing sugars, **(B)** sucrose, and **(C)** starch in different plant parts as a function of sugarcane RB966928 and RB867515 varieties and low and suitable phosphorus (P) levels. Different letters for the same plant part indicate significant differences between sugarcane variety and P level by Student’s *t*-test at *p* ≤ 0.05.

Significant differences in carbohydrate partitioning were observed between the treatments, both in plant parts and the total plant (Supplementary Table S1). Regardless of the sugarcane variety or plant part analyzed, RS and sucrose concentrations were significantly higher under suitable P (Figures 1A,B). Compared with low P, the RS concentrations in the roots, stalks, leaves, and total plant were 19, 36, 42, and 33% higher in RB867515 and 12, 41, 58, and 43% higher in RB966928 under suitable P. Sucrose concentrations in the roots, stalks, leaves, and total plant were 67, 60, 55, and 60% higher in RB867515 and 27, 57, 52, and 51% higher in RB966928 under suitable P.

Conversely, starch concentrations were higher under low P (Figure 1C). In addition, starch concentrations were significantly higher in RB867515 than in RB966928 in all parts of the plant under suitable P and in the roots and stalks under low P. Compared with suitable P, starch concentrations in the roots, stalks, leaves, and total plant were 27, 34, 26, and 28% higher in RB867515 and 57, 64, 71, and 65% higher in RB966928 under low P.

### Antioxidant Enzyme Activities and Proline and Protein Concentrations

**Figure 2:**
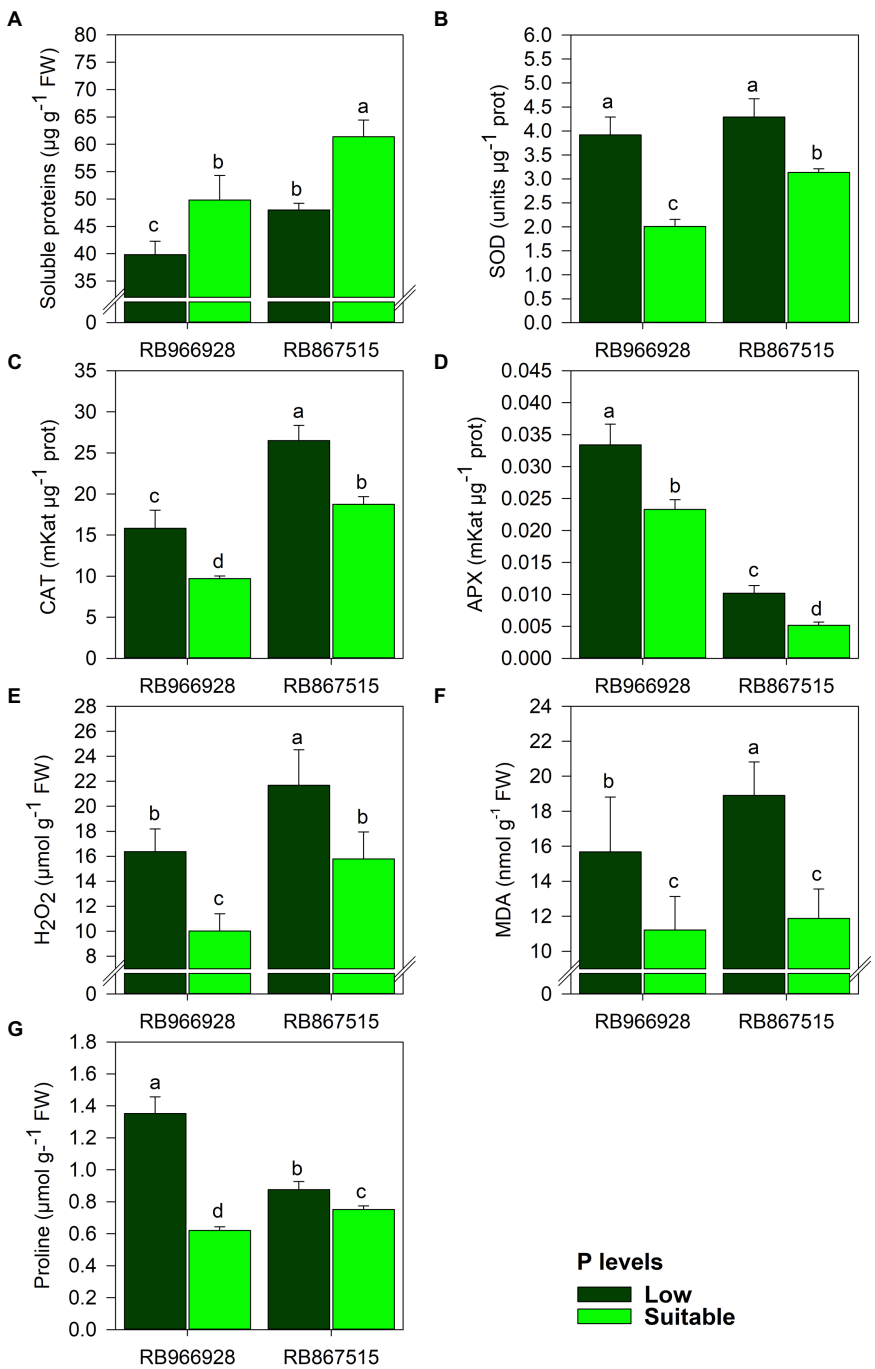
Leaf levels of **(A)** soluble sugars, **(B)** superoxide dismutase (SOD) activity, **(C)** catalase (CAT) activity, **(D)** ascorbate peroxidase (APX) activity, **(E)** hydrogen peroxide (H_2_O_2_), **(F)** malondialdehyde (MDA), and **(G)** proline as a function of sugarcane RB966928 and RB867515 varieties and low and suitable P levels. Different letters indicate significant differences between sugarcane variety and P level by Student’s *t*-test at *p* ≤ 0.05. The error bars express the SE of the mean (*n* = 5).

As shown in Supplementary Table S2, significant differences in antioxidant enzyme activities and proline and protein concentrations were observed between both P levels and varieties (Supplementary Table S2). Compared with suitable P, soluble protein content decreased by 25 and 28% in RB966928 and RB867515, respectively, under low P (Figure 2A). Conversely, compared with suitable P, low P increased the activities of the antioxidant enzymes SOD (by 95% in RB966928 and 56% in RB867515), CAT (by 63% in RB966928 and 67% in RB867515), and APX (by 43% in RB966928 and 220% in RB867515) and the concentrations of H_2_O_2_ (by 63% in RB966928 and 32% in RB867515), MDA (by 40% in RB966928 and 20% in RB867515), and proline (by 120% in RB966928 and 54% in RB867515; Figures 2B–G).

### Nutritional Status

**Figure 3:**
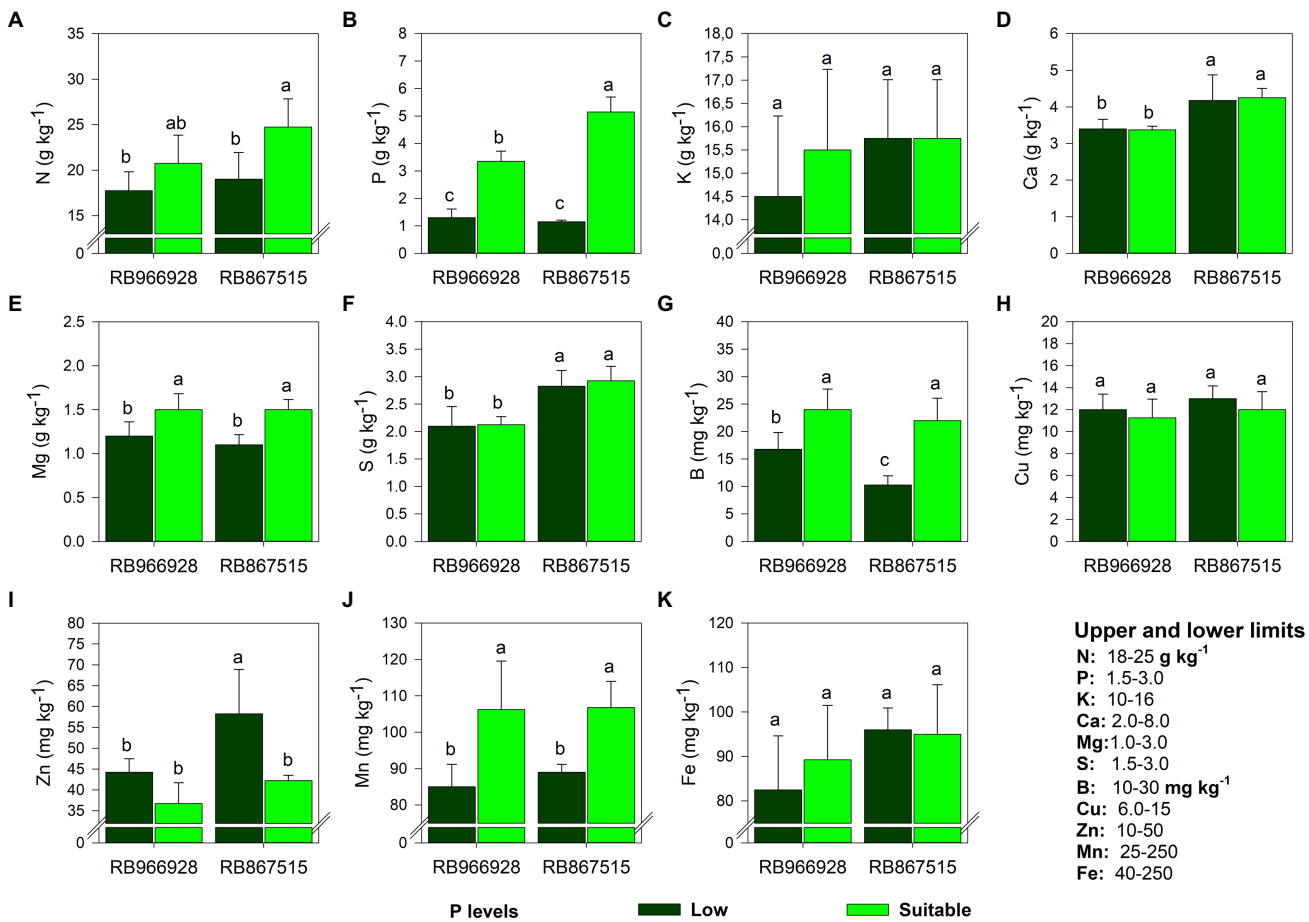
Leaf concentration of **(A)** N, **(B)** P, **(C)** K, **(D)** Ca, **(E)** Mg, **(F)** S, **(G)** B, **(H)** Cu, **(I)** Zn, **(J)** Mn, and **(K)** Fe as a function of sugarcane RB966928 and RB867515 varieties and low and suitable P levels. Different letters indicate significant differences in sugarcane variety and P level by Student’s *t*-test at *p* ≤ 0.05. The error bars express the SE of the mean (*n* = 5).

Except for K, Cu, and Fe, significant effects of the treatments on all analyzed elements were observed (Supplementary Table S3). The leaf concentrations of Ca and S were affected by variety but not P level (Figure 3). The concentrations of all elements were within the acceptable ranges proposed for sugarcane in all treatments (Spironello et al., 1996; Figure 3). Compared with low P, suitable P increased the leaf concentrations of N (by 29% in RB867515, Figure 3A), P (by 160% in RB966928 and 350% in RB867515, Figure 3B), Mg (by 26% in RB966928 and 37% in RB867515, Figure 3E), B (by 118% in RB966928 and 42% in RB867515, Figure 3G), and Mn (by 25% in RB966928 and 20% in RB867515, Figure 3J). Suitable P decreased leaf Zn concentrations by 38% in RB867515, but the concentration of Zn remained within the acceptable range proposed for sugarcane (Spironello et al., 1996; Figure 3I).

### Growth Parameters

**Figure 4:**
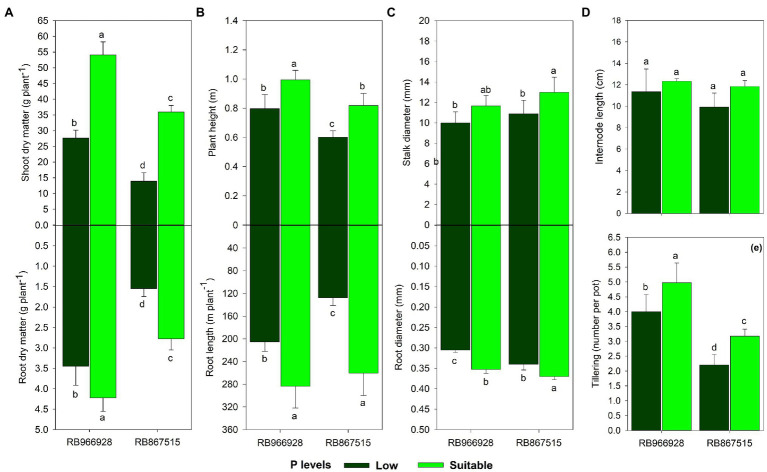
Biometrics of **(A)** root and shoot dry matter, **(B)** root length and plant height, **(C)** root and stalk diameter, **(D)** internode length, and **(E)** tillering as a function of sugarcane RB966928 and RB867515 varieties and low and suitable P levels. Different lower-case letters indicate significant differences in sugarcane variety and P level interaction by Student’s *t*-test at *p* ≤ 0.05. The error bars express the SE of the mean (*n* = 5).

All growth parameters except internode length were influenced by the treatments (Supplementary Table S4). Under suitable P, root and shoot dry matter increased by 23 and 96%, respectively, in RB966928 and by 80 and 160% in RB867515. Root dry matter was 120 and 53% higher in RB966928 than in RB867515 under low and suitable P, respectively, and shoot dry matter was 99 and 51% higher in RB966928 than in RB867515 under low and suitable P, respectively (Figure 4A).

Compared with low P, plant height was 25 and 36% greater under suitable P in RB966928 and RB867515, respectively, and root length was 38 and 104% greater in these varieties. In addition, under low and suitable P levels, plant height was 32 and 61% greater, respectively, in RB966928 than in RB867515, and root length was 61 and 8% greater in RB966928 than in RB867515 (Figure 4B).

Compared with low P, root and stalk diameters were 15 and 16% higher, respectively, in RB966928 and 8 and 19% higher in RB867515 under suitable P. Only root diameter differed significantly between the varieties and was 12 and 5% higher in RB867515 under low and suitable P, respectively (Figure 4C).

Under suitable P, tillering increased by 24 and 44% in RB966928 and RB867515, respectively. Under low and suitable P, tillering was 82 and 57% greater, respectively, in RB966928 than in RB867515 (Figure 4E).

### Principal Component Analysis

**Figure 5:**
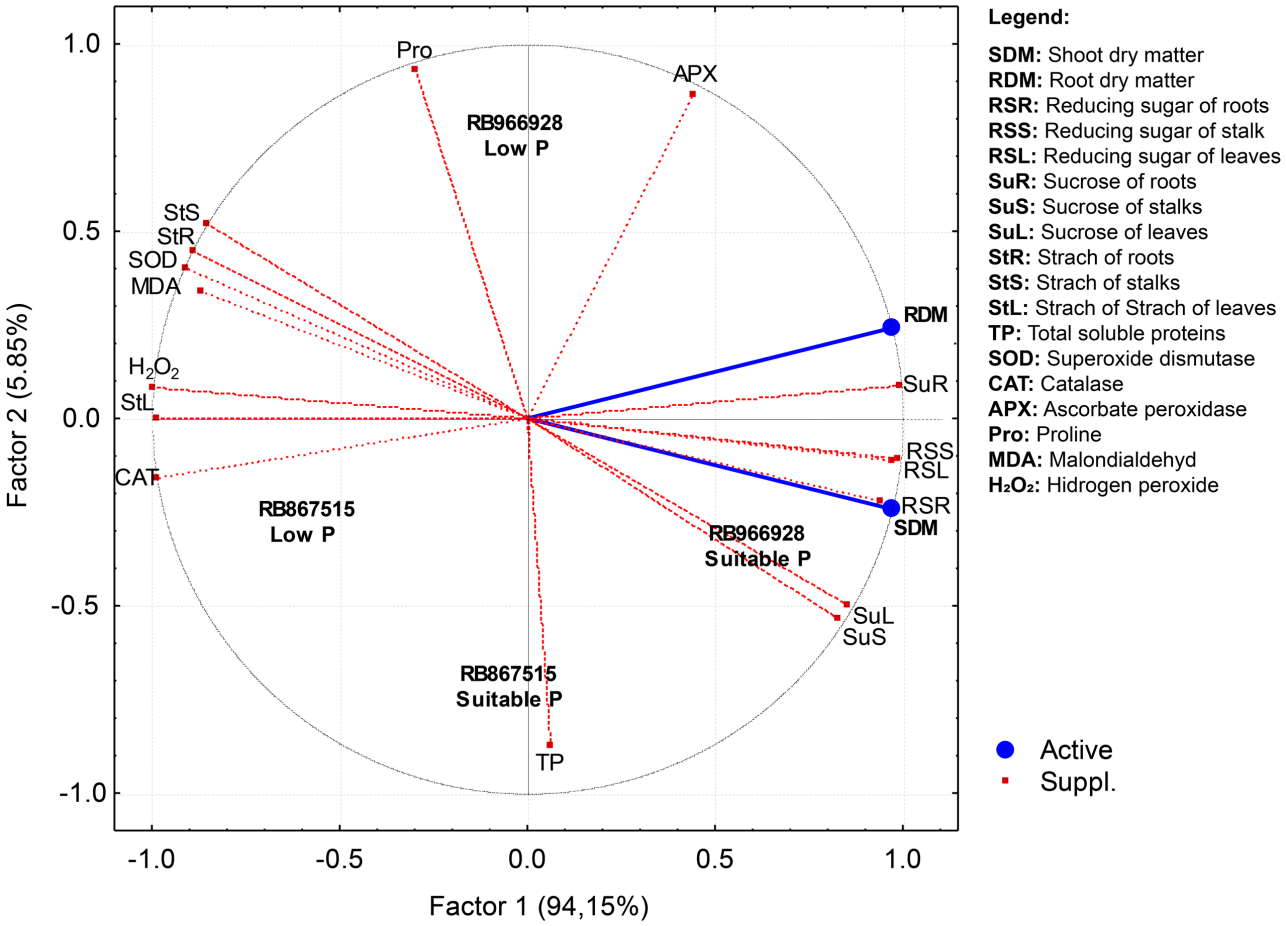
Projection of dataset based on correlations between P levels and sugarcane varieties subjected to principal component analysis (PCA).

Principal component analysis was performed to identify the attributes responsible for the variations in root and shoot dry matter according to P level and variety (Figure 5). Only factor 1 (horizontal axis) presented eigenvalues ≥ 1, and thus factor 2 (vertical axis) was not considered (Table 1). Factor 1 explained 94.15% of the variation in root and shoot dry matter. The RS contents of roots, stalks, and leaves and the sucrose contents of roots, stalks, and leaves were the most important parameters responsible for higher root and shoot dry matter production and were influenced by the variety RB966928 under suitable P. Total soluble proteins explained the influence of variety RB867515 on root and shoot dry matter production under suitable P. By contrast, starch concentrations in roots, stalks, and leaves, the activities of the antioxidant scavenging enzymes SOD and CAT, and H_2_O_2_ and MDA content negatively influenced root and shoot dry matter production and were correlated with low P, regardless of sugarcane variety.

**Table 1:** Principal component analysis results for plant biometric, metabolites, antioxidant enzymes, and amino acid and their correlations with sugarcane root and shoot dry matter.

	Eigenvalue	Total variance %	Cumulative eigenvalues	Cumulative %
1	1.88	94.15	1.88	94.15
				
2	0.12	5.85	2.00	100.00
	Eigenvectors	Correlations
Active	Factor 1	Factor 2	Factor 1	Factor 2
Shoot dry matter	0.7	−0.7	0.97	−0.24
Root dry matter	0.7	0.7	0.97	0.24
	Correlations between soil properties^1^
Supplementary	Factor 1	Factor 2		
RS of roots	0.94	−0.22		
RS of stalks	0.98	−0.10		
RS of leaves	0.97	−0.11		
Sucrose of roots	0.99	0.08		
Sucrose of stalks	0.82	−0.53		
Sucrose of leaves	0.85	−0.49		
Starch of roots	−0.89	0.44		
Starch of stalks	−0.85	0.52		
Starch of leaves	−0.98	−0.01		
Total soluble proteins	0.05	−0.87		
SOD^2^	−0.91	0.40		
CAT^3^	−0.98	−0.15		
APX^4^	0.44	0.86		
Proline	−0.30	0.93		
H_2_O_2_^5^	−0.99	0.08		
MDA^6^	−0.87	0.34		

1 Correlations ≥ |0.70| are significant (Manly, 1994).

2 Superoxide dismutase.

3 Catalase.

4 Ascorbate peroxidase.

5 Hydrogen peroxidase.

6 Malondialdehyde.

## Discussion

### Carbohydrate Partitioning and Antioxidant Molecules

The partitioning of carbohydrates was directly affected by P levels and was highly correlated with root and shoot dry matter yield according to PCA. The synthesis of RS and sucrose is directly proportional to photosynthetic activity and carbohydrate transport (positive feedback; Wang et al., 2013). Due to the relationship between source and sink organs, a high demand for sugars for oxidative processes is expected since every energy-dependent reaction in plant metabolism requires suitable P concentrations (Taiz and Zeiger, 2017). Conversely, the starch concentration increased under low P levels due to the role of P in the regulation of metabolic pathways and sugar transport. Sugar transport into the cytoplasm, where sucrose is synthesized, requires a certain concentration of P in the chloroplast (Cakmak et al., 1994). Therefore, under P deficiency, sugars metabolized in the Calvin cycle remain stored as starch in the stroma of chloroplasts (Fagan et al., 2016). Moreover, stress conditions potentiate carbohydrate accumulation in roots, which act as a preferential sink for sugars, mainly under P deficiency (Wang et al., 2013).

The relationship between sugar production and storage indicates the sucrose yield efficiency of the sugarcane variety. Varieties that produce more sugar likely have greater photosynthetic capacity and are better adapted to local environmental conditions (Zambrosi et al., 2014; Li et al., 2019). According to PCA, the relationship between RS and sucrose was closer in RB966928 than in RB867515. APX activity appeared to be an important modulator of the effect of P availability on the biometric parameters of the sugarcane varieties: compared with suitable P, low P increased APX activity by 50% in RB867515 and 30% in RB966928. APX plays a key role in ROS scavenging, as even very low activity is sufficient for H_2_O_2_ decomposition (Anjum et al., 2014; Ozyigit et al., 2016). The differences between the varieties may reflect their genetic characteristics governing nutrient exploitation and use, considering the role of P in the activity and conformation of enzymes involved in carbohydrate metabolism and partitioning (Fredeen et al., 1989; He et al., 2017).

Antioxidant activity was greater under low P than under suitable P. Stresses can act directly on photosynthesis by reducing the assimilation of CO_2_ (closure of stomata). MDA is frequently used as a biomarker of oxidative damage to cell membranes. MDA content increased under low P, indicating lipid peroxidation (Choudhury et al., 2017). Another potential marker of oxidative stress levels is cellular H_2_O_2_ content. H_2_O_2_ is an active signaling molecule involved in different cellular responses, such as regulation of plant growth, but high cytosolic concentrations of H_2_O_2_ can cause cell damage (Sofo et al., 2015). The increases in plant MDA and H_2_O_2_ contents under low P suggest the presence of stress due to lipid peroxidation of cell membranes.

Consistent with the MDA and H_2_O_2_ results, the activities of the scavenging enzymes SOD, CAT, and APX were increased in plants grown under low P. PCA also demonstrated an inverse relationship between scavenging enzyme activity and root and shoot yields, which were reduced under low P. SOD is the first line of defense against oxidative stress. SOD dismutates H_2_O_2_ into the less toxic molecule O_2_ (del Río et al., 2018). CAT and APX convert H_2_O_2_ into H_2_O (Leung, 2018; Maruta and Ishikawa, 2018). The activities of these enzymes are elevated in highly stressed plants, such as those under low P. Conversely, under suitable P, the photosynthetic apparatus is capable of consuming any excess energy due to its high efficiency, which reduces ROS production; consequently, the activities of scavenging enzymes are lower in plants under low stress.

The concentration of proline also increased in plants under low P, which reflects the recovery strategy of the plant to transport and use N under stress conditions (Rodrigues et al., 2021). Elevating proline concentrations are also important for maintaining cell turgidity, stomatal opening and increasing the rate of photosynthesis (Hayat et al., 2012). The variety RB966928 appears to have a high capacity for proline upregulation under abiotic stress, which may explain the higher biomass accumulation of this variety compared with RB867515. Higher proline expression capacity reduces the need for protein degradation by plants for ROS scavenging in response to abiotic stresses (Ben Rejeb et al., 2014).

The synthesis of total soluble proteins is controlled by the phosphorylation or dephosphorylation of enzymes, which relies on phosphates from ATP and explains the higher concentration of total protein in plants under suitable P (Sharma and Dubey, 2019). With respect to adaptation to adverse conditions, RB867515 exhibited a greater capacity to upregulate protein synthesis, whereas the osmoregulator proline was present at higher levels in RB966928.

### Plant Nutritional and Biometric Parameters

Synergistic, neutral, and antagonistic effects of suitable P on the uptake of other elements were observed. The positive effects of suitable P on plant nutrition were mainly due to greater root development, the energy dependence of active absorption, and increased transport of elements from roots to leaves (Penn and Camberato, 2019). An adequate concentration of P is also essential for the activation of enzymes involved in plant metabolism, such as nitrate reductase and Mg-ATPase, and the assimilation of nutrients required for energy transfer (Fageria and Moreira, 2011).

Only Zn content was lower under suitable P. Zn is absorbed by plants as a cation (Zn^2+^), whereas P is absorbed as an anion (H_2_PO_4_^−^ or HPO_4_^2−^). These opposing charges can induce P-Zn ionic bond formation [Zn_3_(PO_4_)_2_] in soil and plants, resulting in potential P-induced Zn deficiency (Fageria and Moreira, 2011). Soil–plant dynamics were not examined in this study, but the decrease in Zn content under suitable P was likely due to plant dilution effects (i.e., the increase in plant volume under suitable P) and reduced translocation of Zn from roots to shoots (due to binding of Zn to cell walls or chelation by organic ligands; Barben et al., 2010, 2011; Sacristán et al., 2018; Ghobadi et al., 2020). Although Zn plays a fundamental role in sugarcane tillering and internode length (Crusciol et al., 2021), these parameters were not affected by suitable P, and leaf Zn concentrations were within the range proposed for sugarcane in all treatments (Spironello et al., 1996). The differences in Zn content between the varieties are mainly due to differences in genetic characteristics and adaptive strategies that enhance root exploration to ensure adequate P uptake (Smith et al., 2005).

All biometric parameters except internode length increased under suitable P compared with low P (Figures 4A–D). Plant growth and development are highly P dependent due to the direct participation of P in energy-related metabolic pathways, cell division and elongation, photosynthesis, cellular respiration, organic compound synthesis, ionic absorption, and growth hormone synthesis (Sun et al., 2016). Suitable P positively influenced photosynthetic parameters, carbohydrate synthesis and partitioning, nutritional status, and below- and aboveground biometric parameters. The variety RB966928 performed better than RB867515 in most parameters, indicating greater adaptive capacity of RB966928 regardless of P conditions. The plasticity and environmental adaptability of varieties can govern biometric characteristics such as root mass diameter and quantity and P absorption capacity (Sun et al., 2016, 2017). In general, differences between varieties are mainly dictated by genetic attributes, such as the rate of metabolic processes, hormonal regulation, and meristematic cell division and elongation (Zambrosi et al., 2014). Nonetheless, suitable P led to better yield performance regardless of the sugarcane variety.

## Conclusion

This study assessed the physiological and nutritional attributes of two sugarcane varieties to evaluate the efficiency of their responses to conditions of low and adequate P concentrations. Most attributes were directly influenced by P levels, including sugarcane yield. Antioxidant activity in response to adverse conditions, i.e., low P supply, was observed in both varieties, but RB966928 was more sensitive to low P levels and more responsive to P supply than RB867515. RB867515 appears to use the antioxidant enzyme ascorbate reductase to overcome P limitation, whereas RB966928 relies on increased levels of proline to increase the efficiency of photoassimilate accumulation. Understanding these characteristics can facilitate sugarcane crop management and variety selection, especially under conditions in which P is the most limiting nutrient.

## Data Availability Statement

The datasets presented in this study can be found in online repositories. The names of the repository/repositories and accession number(s) can be found in the article/Supplementary Material.

## Author Contributions

CC, MT, RS, and JM designed the experiment. MT, RS, and JM obtained and process the data. MC, LM, CN, and AG analyzed the data and wrote the manuscript with contributions of all co-authors. All authors contributed to the article and approved the submitted version.

## Conflict of Interest

The authors declare that the research was conducted in the absence of any commercial or financial relationships that could be construed as a potential conflict of interest.

## Publisher’s Note

All claims expressed in this article are solely those of the authors and do not necessarily represent those of their affiliated organizations, or those of the publisher, the editors and the reviewers. Any product that may be evaluated in this article, or claim that may be made by its manufacturer, is not guaranteed or endorsed by the publisher.
