# Supplementary material for: Carbohydrate Partitioning and Antioxidant Substances Synthesis Clarify the Differences Between Sugarcane Varieties on Facing Low Phosphorus Availability
Source: Front Plant Sci. 2022 May 11;13:888432. doi: 10.3389/fpls.2022.888432 (PMC9131043; doi:10.3389/fpls.2022.888432)
Supplement: Supplementary file 1 [file Data_Sheet_1.docx]

Supplementary Material

Table S1: P values indicating the potential effect of sugarcane varieties and P levels on carbohydrates concentration and partitioning.

| Carbohydrates | Plant parts | P value | CV (%) |
| --- | --- | --- | --- |
| Reducing Sugars | Root | <0.001 | 5.08 |
|  | Stalk | <0.001 | 10.11 |
|  | Leaf | <0.001 | 9.96 |
|  | Total | <0.001 | 9.19 |
| Sucrose | Root | <0.001 | 9.60 |
|  | Stalk | <0.001 | 11.29 |
|  | Leaf | <0.001 | 11.26 |
|  | Total | <0.001 | 9.97 |
| Starch | Root | <0.001 | 6.98 |
|  | Stalk | <0.001 | 9.43 |
|  | Leaf | <0.001 | 9.85 |
|  | Total | <0.001 | 5.65 |

Table S2: P values indicating the potential effect of sugarcane varieties and P levels on soluble proteins, proline, hydrogen peroxide, malondialdehyde (MDA), and activities of scavenging antioxidant enzymes (SOD, CAT, and APX).

| P probability | Soluble proteins | SOD | CAT | APX | H_2_O_2_ | MDA | Proline |
| --- | --- | --- | --- | --- | --- | --- | --- |
| P value | <0.001 | <0.001 | <0.001 | <0.001 | <0.001 | <0.001 | <0.001 |
| CV (%) | 6.12 | 8.33 | 8.62 | 10.59 | 13.32 | 15.47 | 6.70 |

Table S3: P values indicating the potential effect of sugarcane varieties and P levels on the nutrient leaves’ concentration.

| Elements | P probability |  |
| --- | --- | --- |
|  | P value | CV (%) |
| N | 0.0224 | 13.77 |
| P | <0.001 | 13.38 |
| K | 0.6164 | 9.84 |
| Ca | 0.0109 | 10.42 |
| Mg | 0.0037 | 11.10 |
| S | <0.001 | 10.99 |
| B | <0.001 | 18.00 |
| Cu | 0.2048 | 10.20 |
| Zn | 0.0022 | 13.48 |
| Mn | 0.0041 | 8.51 |
| Fe | 0.2926 | 11.61 |

Table S4: P values indicating the potential effect of sugarcane varieties and P levels on biometric parameters of sugarcane root and shoot.

| Parameters |  | P-value | CV% |
| --- | --- | --- | --- |
| Dry matter | Root | <0.001 | 11.03 |
|  | Shoot | <0.001 | 9.01 |
| Biometry | Root length | <0.001 | 2.89 |
|  | Plant heigh | <0.001 | 9.37 |
|  | Internode length | 0.0984 | 11.27 |
|  | Tillering | <0.001 | 13.59 |
| Diameter | Root | <0.001 | 2.89 |
|  | Shoot | 0.0306 | 10.94 |
